# Spatiotemporal cytokinin response imaging and ISOPENTENYLTRANSFERASE 3 function in Medicago nodule development

**DOI:** 10.1093/plphys/kiab447

**Published:** 2021-09-21

**Authors:** Paolo M Triozzi, Thomas B Irving, Henry W Schmidt, Zachary P Keyser, Sanhita Chakraborty, Kelly Balmant, Wendell J Pereira, Christopher Dervinis, Kirankumar S Mysore, Jiangqi Wen, Jean-Michel Ané, Matias Kirst, Daniel Conde

**Affiliations:** 1 School of Forest, Fisheries and Geomatics Sciences, University of Florida, Gainesville, Florida 32611, USA; 2 Department of Bacteriology, University of Wisconsin-Madison, Madison, Wisconsin 53706, USA; 3 Noble Research Institute, Ardmore, Oklahoma 73401, USA; 4 Department of Agronomy, University of Wisconsin-Madison, Madison, Wisconsin 53706, USA; 5 Genetics Institute, University of Florida, Gainesville, Florida 32611, USA

## Abstract

Most legumes can establish a symbiotic association with soil rhizobia that trigger the development of root nodules. These nodules host the rhizobia and allow them to fix nitrogen efficiently. The perception of bacterial lipo-chitooligosaccharides (LCOs) in the epidermis initiates a signaling cascade that allows rhizobial intracellular infection in the root and de-differentiation and activation of cell division that gives rise to the nodule. Thus, nodule organogenesis and rhizobial infection need to be coupled in space and time for successful nodulation. The plant hormone cytokinin (CK) contributes to the coordination of this process, acting as an essential positive regulator of nodule organogenesis. However, the temporal regulation of tissue-specific CK signaling and biosynthesis in response to LCOs or *Sinorhizobium meliloti* inoculation in *Medicago truncatula* remains poorly understood. In this study, using a fluorescence-based CK sensor (*pTCSn::nls:tGFP*), we performed a high-resolution tissue-specific temporal characterization of the sequential activation of CK response during root infection and nodule development in *M. truncatula* after inoculation with *S. meliloti*. Loss-of-function mutants of the CK-biosynthetic gene *ISOPENTENYLTRANSFERASE 3* (*IPT3*) showed impairment of nodulation, suggesting that IPT3 is required for nodule development in *M. truncatula*. Simultaneous live imaging of *pIPT3::nls:tdTOMATO* and the CK sensor showed that *IPT3* induction in the pericycle at the base of nodule primordium contributes to CK biosynthesis, which in turn promotes expression of positive regulators of nodule organogenesis in *M. truncatula*.

## Teaser

Precise spatial and temporal characterization of cytokinin (CK) responses reveals the function of the CK biosynthesis gene *ISOPENTENYLTRANSFERASE 3* during nodule development in *Medicago truncatula.*

## Introduction

Legume species acquired the capacity to interact symbiotically with rhizobium bacteria to fix atmospheric dinitrogen, allowing their growth without fertilizers on nitrogen-deprived soils. This interaction involves the development of specific organs, the root nodules, that host rhizobia and provide them with carbon sources and the microenvironment required for nitrogen fixation, a reaction catalyzed by the bacterial enzyme nitrogenase. In most rhizobium–legume associations, the perception of bacterial lipo-chitooligosaccharides (LCOs) in the epidermis, commonly known as Nod factors, initiates a signaling cascade that is transmitted to the inner cell layers activating cell division, with simultaneous rhizobial infection of the host root. In *Medicago truncatula*, rhizobial infection is initiated within a curled root hair tip and the subsequent formation of a transcellular apoplastic compartment called the infection thread (IT). The IT traverses the epidermis, cortex, and ramifies within the confines of the nodule primordium, developed by organized cell divisions in the root endodermis, cortex, and pericycle (Roy et al., 2020). These coordinated mechanisms of root infection and nodule organogenesis ensure that nodule maturation occurs in perfect coordination with nodule colonization by rhizobia (Xiao et al., 2014). *M.* *truncatula* produces indeterminate nodules characterized by a longitudinal gradient of differentiation with a persistent distal apical meristem and older proximal layers (Ferguson et al., 2010). Like other developmental processes, nodulation is modulated by phytohormones (Buhian and Bensmihen, 2018).

The plant hormone cytokinin (CK) is involved in various aspects of plant growth and development. CK signaling consists of a phosphorelay mediated by a two-component system comprising a sensor and a response regulator (RR). The site of CK perception is thought to be the lumen of the endoplasmic reticulum. The CK-induced phosphorelay causes transcriptional changes in the nucleus mediated by type-B and type-A RRs, which play positive and negative roles in this regulation, respectively (Kieber and Schaller, 2018). Type-B RRs typically bind to target genes at the consensus sequence (A/G)GAT(T/C) enriched in their *cis-*regulatory regions. Synthetic CK sensors called two-component signaling sensors (TCS) containing concatemeric versions of this sequence have been developed for plants (Zürcher et al., 2013). CK plays essential role during nodule formation (Ferguson and Mathesius, 2014; Gamas et al., 2017). In *M. truncatula*, CK accumulates in the section of the root susceptible to rhizobial infection as early as 3 h after LCO treatment (van Zeijl et al., 2015). The TCS is activated by rhizobium in the cortical cells of *M. truncatula* that go on to form indeterminate nodules, and those in *Lotus japonicus* which form determinate nodules (Held et al., 2014; Jardinaud et al., 2016). In soybean (*Glycine max*), a regulatory feedback loop involving auxin and CK governs proper determinate nodule development (Turner et al., 2013). Rhizobia also induce the expression of CK biosynthetic and signaling genes in the epidermis of *M. truncatula* (Liu et al., 2015; Damiani et al., 2016; Jardinaud et al., 2016). The *pMtRR9::GUS* transcriptional reporter, a CK RR type-A (RRA), was rapidly detected in the root epidermis, in addition to other root tissues, in response to LCOs (Op den Camp et al., 2011). A more recently developed CK signaling sensor termed TCS new (TCSn) (Zürcher et al., 2013), driving GUS expression, enabled detection of the activation of a CK response in the *M. truncatula* root epidermis and the outer cortex 8 h after the LCO treatment or *Sinorhizobium* *meliloti* inoculation (Jardinaud et al., 2016). In *L. japonicus*, the *TCS* reporter was activated first in the cortex and only later in the epidermis by rhizobia (Held et al., 2014; Reid et al., 2017). The differing sequence of activation of CK responses during early symbiotic stages is likely a reflection of differences in the process of nodule development between them (Gamas et al., 2017).

CK plays an antagonistic role during root infection at the epidermis and nodule formation in the cortex (Gamas et al., 2017). The positive regulation of CK on nodule formation was first reported by physiological studies, which showed that exogenous CK induces nodule-like structures on the roots of several legumes (Heckmann et al., 2011). Further evidence for the positive role of CK in nodule inception (NIN) has come from the analysis of nodulation-defective mutants altered in CK receptors, LOTUS HISTIDINE KINASE 1 (LHK1) in *L. japonicus*, and CK RESPONSE 1 (CRE1) in *M. truncatula* (Gonzalez-Rizzo et al., 2006; Murray et al., 2007; Tirichine et al., 2007; Plet et al., 2011), and other CK receptors in both legumes (Held et al., 2014; Boivin et al., 2016). Moreover, a gain-of-function *LHK1* line generates spontaneous nodules in the absence of the rhizobia (Tirichine et al., 2007; Madsen et al., 2010). Transcriptomic analyses in *M. truncatula* identified symbiotic genes that are rapidly induced by exogenous CK on roots (Ariel et al., 2012), such as *NIN*. NIN and the CRE1-dependent pathways are connected by a positive feedback loop, with NIN binding to the *CRE1* promoter and activating its expression (Vernié et al., 2015). Similarly, *CRE1* is required for CK-induced *NIN* expression (Plet et al., 2011). In *L. japonicus* roots, exogenous CK treatment also induces *NIN* specifically in root cortical cells (Heckmann et al., 2011). Moreover, *NIN* ectopic expression leads to root cortical cell divisions and nodule-like structures in both *L. japonicus* and* M. truncatula* (Soyano et al., 2013; Vernié et al., 2015), through the activation of transcription factors, such as *NUCLEAR FACTOR Y SUBUNIT A1 (NF-YA1)* and* B1 (NF-YB1)* (Soyano et al., 2013; Laloum et al., 2014; Hossain et al., 2016; Shrestha et al., 2021). In contrast, in the epidermis of *M. truncatula*, CK negatively regulates root infection (Gamas et al., 2017). Indeed, depletion of the epidermal CK pool obtained by expressing a *CK OXIDASE/DEHYDROGENASE* enzyme under an epidermis-specific promoter caused an increased number of ITs and nodules (Jardinaud et al., 2016). Additionally, exogenous CK treatment inhibited the induction of the LCO response and pre-infection marker *EARLY NODULIN 11*, in a *MtCRE1*-dependent fashion (Jardinaud et al., 2016). Recently, a link between the epidermis-derived CK and cortical cell divisions was established (Jarzyniak et al., 2021). *M.* *truncatula* ATP-binding cassette transporter 56 (MtABCG56) transports CK from the epidermal to cortical cells, activating the CRE1-dependent CK responses, including the *RRA4* (Jarzyniak et al., 2021). These downstream responses trigger further CK biosynthesis required for nodule development (Mortier et al., 2014; van Zeijl et al., 2015; Vernié et al., 2015).

Several CK biosynthesis genes, including *ISOPENTENYLTRANSFERASE 3* (*IPT3*) and *IPT1*, *CYP735A1*, *LONELY GUY 1* (*LOG1*), and *LOG2*, are upregulated in response to LCOs or during nodulation in *L. japonicus* and* M. truncatula* (Chen et al., 2014; Mortier et al., 2014; Azarakhsh et al., 2015; van Zeijl et al., 2015; Reid et al., 2017; Azarakhsh et al., 2018; Schiessl et al., 2019). The expression of *MtIPT3*, *MtLOG1*, and *MtLOG2* transcriptional GUS reporters was also detected in the nodule primordium (Mortier et al., 2014; Azarakhsh et al., 2020). Decreasing *LOG1* expression leads to impaired nodulation in *M. truncatula* (Mortier et al., 2014). All these studies highlight the importance of CK biosynthesis during root infection and nodule development. Transcriptional fusions using GUS gene reporter allowed identifying CK signaling at a tissue-specific level in *M. truncatula* roots during these biological processes. However, these studies have been limited in their temporal resolution. A detailed spatial and temporal characterization of the CK response in *M. truncatula* roots should clarify the role of this hormone in nodule induction and organogenesis.

In this study, we present the spatiotemporal regulation of CK response in rhizobia-inoculated roots using a fluorescence-based CK signaling sensor, *pTCSn::nls:tGFP*. To further explore the potential of this sensor, we employed it along with the transcriptional fusion of the CK biosynthetic gene *IPT3* during nodule development. Simultaneous monitoring of the *pIPT3::nls:tdTOMATO* reporter and the CK sensor activities during nodule development suggested that *IPT3* induction in the pericycle at the base of nodule primordium contributes to CK biosynthesis, which in turn promotes nodule organogenesis in *M. truncatula*. Furthermore, we analyzed the loss-of-function mutant of *ipt3* and found that it is required for nodule development in *M. truncatula*.

## Results

### A fluorescent protein-based CK sensor is activated in root epidermal and cortical cells upon CK treatment in *M. truncatula*

CK responses have been studied in response to LCOs and *S. meliloti* in *M. truncatula* roots, using transcriptional reporters with *RRAs* or the synthetic *TCSn* promoters fused to the *GUS* gene (Op den Camp et al., 2011; Plet et al., 2011; Jardinaud et al., 2016; Fonouni-Farde et al., 2017). In soybean, fluorescent protein-based auxin and CK transcriptional reporters have been successfully used to monitor and determine their cellular level ratios in root and nodule tissues (Fisher et al., 2018). In *L. japonicus*, a tissue-specific time-course experiment following the activity of *pTCSn::nls:GFP* showed that CK response occurs in cortical cells before expanding to the epidermis (Reid et al., 2017).

In this work, we designed a fluorescent protein-based CK transcriptional reporter that addresses the limitations of the GUS reporter system. This CK sensor consists of the *TCSn* promoter (Zürcher et al., 2013), driving the expression of the turbo green fluorescent protein (tGFP) fused to a nuclear localization signal (nls) peptide (*pTCSn::nls:tGFP*). This alternative approach to the GUS reporter system allows continuous, nondestructive monitoring of CK signaling throughout plant development by live imaging, and co-imaging with other fluorescence-based reporters.

**Figure 1:**
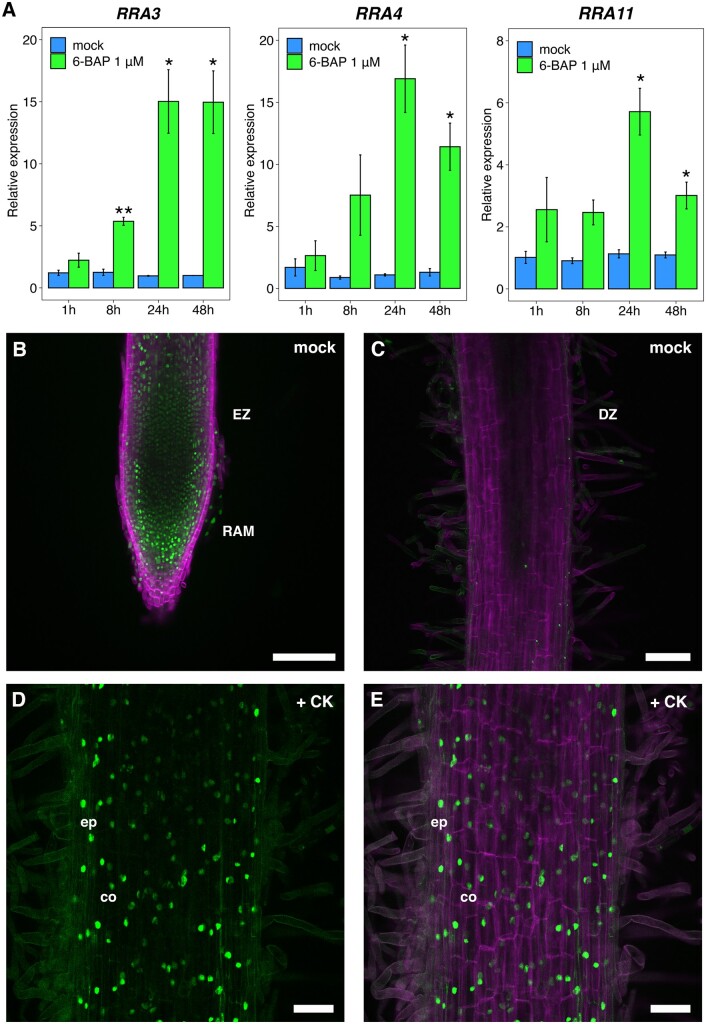
A reporter of CK signaling based on the *pTCSn::nls:tGFP* transcriptional fusion is activated in the epidermal and cortical cells after CK treatment. A, RT-qPCR analyses of *RRAs* gene expression after 1, 8, 24, and 48 h of 1 µM 6-BAP or mock treatment. The Student’s *t* test was performed, and asterisks represent statistically significant differences between 6-BAP and mock treatments in each time point. *P < 0.05, **P < 0.01. Values are the means ± se of fold-changes of two biological replicates (*n* = 2). B, *pTCSn::nls:tGFP* activity in the root tip of nontreated *M. truncatula* transgenic root. The tGFP signal from the nuclei of the RAM and EZ is shown in green. In magenta, the signal emitted by cell wall polysaccharides bound to Calcofluor white M2R is shown. C, *pTCSn::nls:tGFP* activity in the DZ of nontreated transgenic root and (D, E) in DZ of 1 µM 6-BAP treated transgenic root after 24 h in the epidermis (ep) and cortex (co). D, tGFP signal (green) and (E) merged image showing tGFP and Calcofluor white stained (magenta) signals. Scale bar: 100 µm (B and C) and 50 µm (D and E).

Before evaluating the CK sensor activity, we characterized the timing of CK transcriptional responses in *M. truncatula* roots by analyzing the expression profiles of three *RRA* genes, *RRA3*, *RRA4*, and *RRA11*, after 1, 8, 24, and 48 h of 6-benzylaminopurine (6-BAP) treatment. We found that *RRA*s reached their maximum gene expression after 24 h of the 6-BAP treatment (Figure 1A). Based on the time of CK signaling activation, we assessed the tissue-specific CK response using the *pTCSn::nls:tGFP* CK sensor in *M. truncatula* transgenic roots after 24 h of BAP treatment. In nontreated roots, tGFP was primarily detected in the columella, root apical meristem (RAM), and elongation zone (EZ) of the root tip, as previously described for the *pTCSn::GUS* reporter (Figure 1B; Jardinaud et al., 2016; Fonouni-Farde et al., 2017). Very few cells showed nuclei-localized fluorescence in the differentiation zone (DZ) of the root (Figure 1C), indicating the absence of CK response in the DZ in nontreated roots. In contrast, roots treated with 6-BAP for 24 h exhibited a strong signal of nuclei-localized fluorescence in the epidermis and cortex in the DZ of the root (Figure 1, D and E). 4′,6-diamidino-2-phenylindole (DAPI) counterstain confirmed that tGFP fluorescence was localized to the nuclei (Supplemental Figure S1). These observations indicate that the *pTCSn::nls:tGFP* sensor constitutes a suitable molecular tool to investigate the CK response in *M. truncatula* and that CK signal transduction occurs in *M. truncatula* epidermal and cortical cells after the application of CK to the root.

### Tissue-specific time-course of CK signaling during the early symbiotic interaction with *S. meliloti* in *M. truncatula* roots

**Figure 2:**
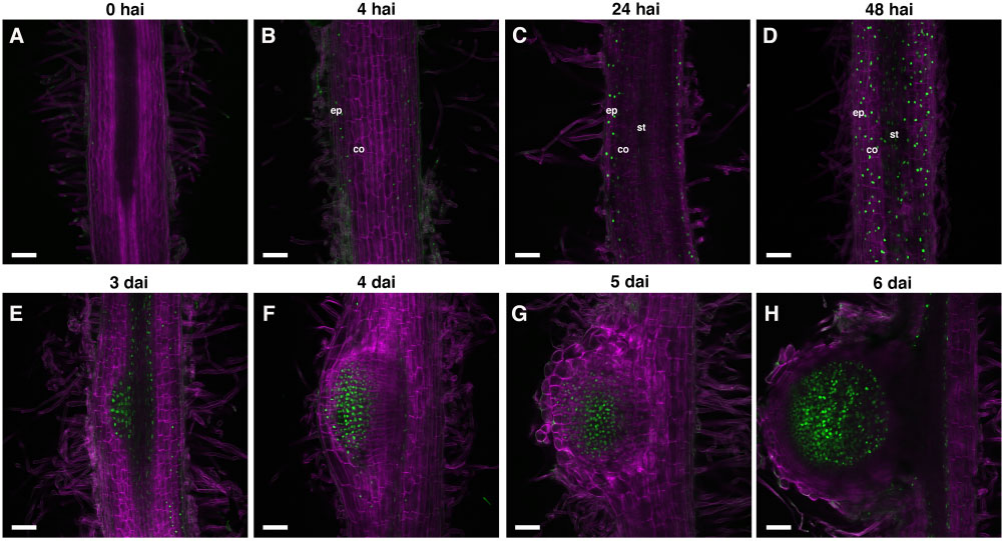
Spatiotemporal activation of CK signaling during indeterminate nodule development in *M. truncatula*. A–D, *pTCSn::nls:tGFP* activity (green) and cell walls (calcofluor white stained, magenta) in the susceptible zone of transgenic root at 0, 4, 24, and 48 hai with *S. meliloti*. Epidermis (ep), cortex (co), and stele (st). E–H, *pTCSn::nls:tGFP* activity during nodule primordium development at (E) Stages II/III, (F) Stages IV/V, (G) Stage VI and (H) mature nodule. Scale bar: 100 µm.

Characterization of CK response during indeterminate nodule development remains limited to a few time points during the process by using *RRA* promoters fused to GUS reporters in *M. truncatula* (Op den Camp et al., 2011; Plet et al., 2011). To obtain a high spatiotemporal resolution of CK response during indeterminate nodule development in *M. truncatula*, we analyzed the activity of *pTCSn::nls:tGFP* in a time-course experiment using transgenic roots after *S. meliloti* inoculation. Prior to inoculation, very low *pTCSn::nls:tGFP* activity was detected in the cell layers of the susceptibility zone (SZ; Figure 2A). At 4 hours after inoculation (hai), *pTCSn::nls:tGFP* activity started in the epidermal cells of the SZ, indicating that rhizobia-induced CK response occurs very early in the epidermis of *M. truncatula* (Figure 2B). At 24 hai, nuclei-localized fluorescence was still observed in the epidermal cells but was also present in outer cortical cells of the SZ (Figure 2C) and by 48 hai, strong fluorescence was widespread in the outer and inner cortical cell layers of the SZ (Figure 2D). Thus, CK signaling is activated first in the epidermis, reaches the outer cortical cells within 24 hai and extends to most cortical cell layers within 48 hai.

### Tissue-specific time-course of CK signaling during the indeterminate nodule development in *M. truncatula*

We also monitored the *pTCSn::nls:tGFP* activity throughout nodule development from the first cell divisions in the pericycle, the endodermis, and the cortex, until the mature nodule formation. Moreover, we associate the tissue-specific *pTCSn::nls:tGFP* activity time-course with the sequential cell division program characterized previously during nodule formation (Xiao et al., 2014).

At 3 d after inoculation (dai), the *pTCSn::nls:tGFP* signal that was widely distributed across the cortical cell layers of the SZ disappears (Figure 2D), giving rise to a robust and more localized signal at the pericycle and dividing cortical cell layers C3–C5. These cells are related with the nodule primordium initiation at the developmental Stages II and III (Figure 2E; Xiao et al., 2014). This nodule primordium-specific pattern of the CK response allowed us to clearly distinguish a nodule primordium from a lateral root primordium (LRP) during their early developmental stages, where tGFP expression was weaker and limited to the vasculature and developing meristem (Supplemental Figure S2). At 4 dai, we found that the Stages IV and V nodule primordia showed the CK signaling activation extending to most of the dividing cortical cell layers (Figure 2F). At 5 dai, the nodule primordium emerges from the main root and becomes a true nodule when the meristem starts functioning (Stage VI). At this point, the CK response was localized to the C3, the C4-to C5-derived cells that form the multi-layered nodule meristem (C3) and the nonmeristem zone immediately below (C4 and C5), respectively (Figure 2G). At 6 dai, the nodule is in an advanced developmental stage, with the vascular bundles starting to surround the nodule meristem. At this stage, the CK signaling was strongly activated in the central zone of the nodule, including the nodule meristem and the C4/5-derived cells that will be colonized by rhizobia (Figure 2H).

### CK biosynthesis by IPT3 is required for nodule development in *M. truncatula*

CK and its downstream responses are critical regulators of nodule initiation and development. However, the molecular mechanism of the local CK biosynthesis during nodule organogenesis remains poorly characterized in *M. truncatula.* It has been proposed that KNOX3 directly activates the transcription of CK biosynthesis genes, *IPT3*, *LOG1*, and *LOG2*, to promote CK biosynthesis during nodule organogenesis (Azarakhsh et al., 2015, 2020). However, the genetic characterization of CK biosynthesis genes in *M. truncatula* remains limited to *LOG1* (Mortier et al., 2014)*. IPT3* expression is induced at 72 hai in the root, reaching a maximum at 5 dai (Schiessl et al., 2019; Supplemental Figure S3). Moreover, the *pIPT3*::*GUS* reporter showed that *IPT3* is expressed in the nodule primordium of *M. truncatula* (Azarakhsh et al., 2020). These observations indicate that IPT3 represents an excellent candidate to investigate the role of CK biosynthesis during nodule organogenesis.

**Figure 3:**
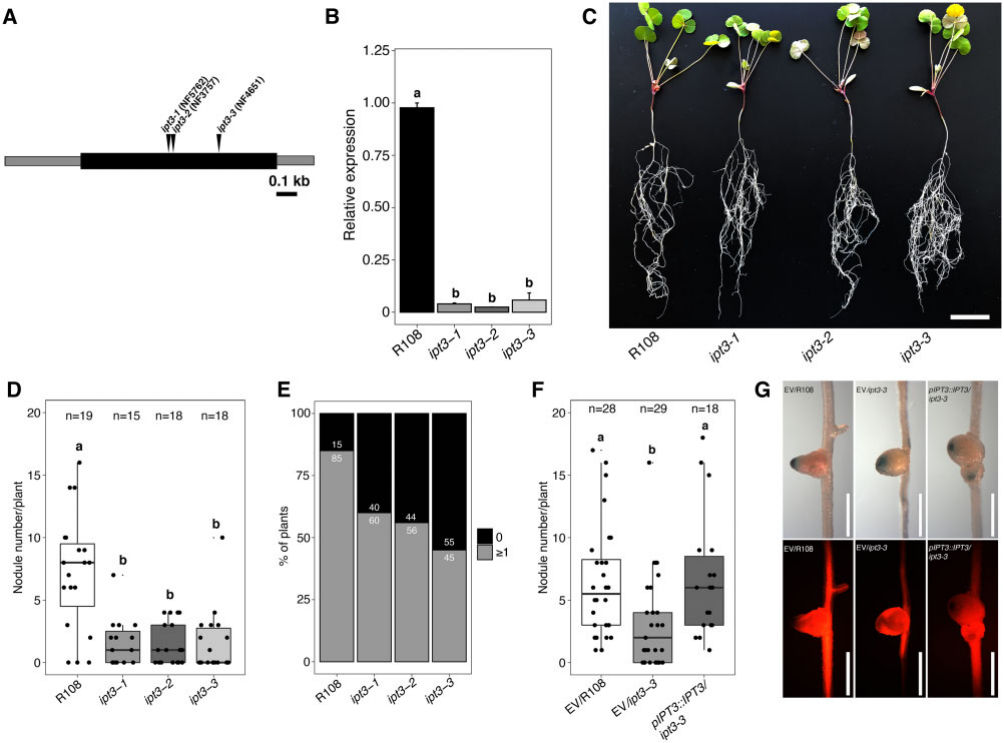
*IPT3* is required for nodule development in *M. truncatula*. A, Schematic diagram showing the genomic *IPT3* gene structure. NF5762, NF3757, and NF4651 *Tnt1* lines have one insertion in the single exon region (black) flanked by 5′- and 3′-untranslated regions (gray) of the gene and were renamed *ipt3-1*, *ipt3-2,* and* ipt3-3*, respectively. Black bar, 100 bp. B, RT-qPCR analysis of *IPT3* gene expression in R108 genotype and *ipt3* homozygous mutants. Values indicate means ± se for three biological replicates (*n* = 3). *P*-values were calculated by analysis of variance (ANOVA) followed by Tukey’s post hoc testing. Groups of significant difference (*P* < 0.05) are indicated with different letters. C, Representative image of 3-week-old R108 and *ipt3* mutant plants at 14 dai with *S. meliloti* 1021. Scale bar: 3 cm. D, Nodule number in R108 and *ipt3* mutants at 14 dai. Boxplots center line, median; the box extends from the 25th to 75th percentiles; whiskers, 1.5 interquartile range; points out of the whiskers, outliers. Statistical analysis was performed using ANOVA followed by Tukey’s post hoc testing. Groups of significant difference (*P* < 0.05) are indicated with different letters. E, Percentage of wild-type (*n* = 19), *ipt3-1* (*n* = 15), *ipt3-2* (*n *= 18), and *ipt3-3* (*n* = 18) mutant plants showing 1 or more nodules or none at 14 dai. F, Number of nodules in transgenic roots of EV/R108, EV/*ipt3-3*, and *pIPT3::IPT3*/*ipt3-3* mutant at 14 dai. Boxplots center line, median; the box extends from the 25–75th percentiles; whiskers, 1.5× interquartile range; points out of the whiskers, outliers. Statistical analysis was performed using ANOVA followed by Tukey’s post hoc test. Groups of significant difference (*P* < 0.05) are indicated with different letters. G, Representative nodules of EV/R108 (*n* = 28), EV/*ipt3-3* (*n* = 29), and *pIPT3::IPT3*/*ipt3-3* (*n* = 18)*.* Blue color indicates that the nodules are infected (upper) and tdTOMATO fluorescence is used to identify the transformed roots (lower). Scale bar: 1 mm.

We identified three *M. truncatula Tnt1* lines showing an insertion in the single exon of *IPT3* (Tadege et al., 2008). These mutants were named *ipt3-1*; *ipt3-2,* and* ipt3-3* (Figure 3A). Isolation of homozygous individuals of *ipt3* was confirmed by reverse transcription quantitative polymerase chain reaction (RT-qPCR) analyses (Figure 3B). Then, *ipt3* mutants and wild-type plants were used to perform nodulation assays. At 14 dai, all the *ipt3* mutants showed a significantly lower nodule number than the control (Figure 3, C and D). Moreover, between 40% and 55% of the *ipt3* mutants did not develop any nodule, compared to 15% in the wild-type background (Figure 3E). Root dry weight measurements revealed no significant differences between wild-type plants and *ipt3* mutants after 14 dai, indicating that the reduction in the number of nodules is not due to defects in root growth (Supplemental Figure S4A), while shoot dry weight significantly decreased only in *ipt3-1* (Supplemental Figure S4B). Cross-sections of wild-type and *ipt3* mutant nodules showed normal structure and colonization at 14 dai (Supplemental Figure S5A). At 21 dai, the number of nodules was reduced in all three mutant backgrounds (Supplemental Figure S5B). These nodules developed normally, and there was no difference in nitrogen fixation in *ipt3* mutant nodules compared with wild-type plants (Supplemental Figure S5C). To validate that the mutation in *IPT3* is causing the defects in nodulation phenotype, we complemented the *ipt3-3* mutant with the functional *IPT3* driven by its native promoter (*pIPT3::IPT3*). At 14 dai, the number of nodules in the *ipt3-3* roots transformed with the empty vector (EV) was significantly lower than the wild-type transformed with the EV. At the same time, no significant differences were observed between *ipt3-3* roots transformed with the *pIPT3::IPT3* construct and wild-type transformed with the EV (Figure 3, F and G), confirming that functional *IPT3* rescues the nodulation defects observed in the *ipt3-3* background and showing that CK biosynthesis by *IPT3* is required for nodulation in *M. truncatula*.

### *IPT3* expression is induced in the stele at the base of the developing nodule primordium

**Figure 4:**
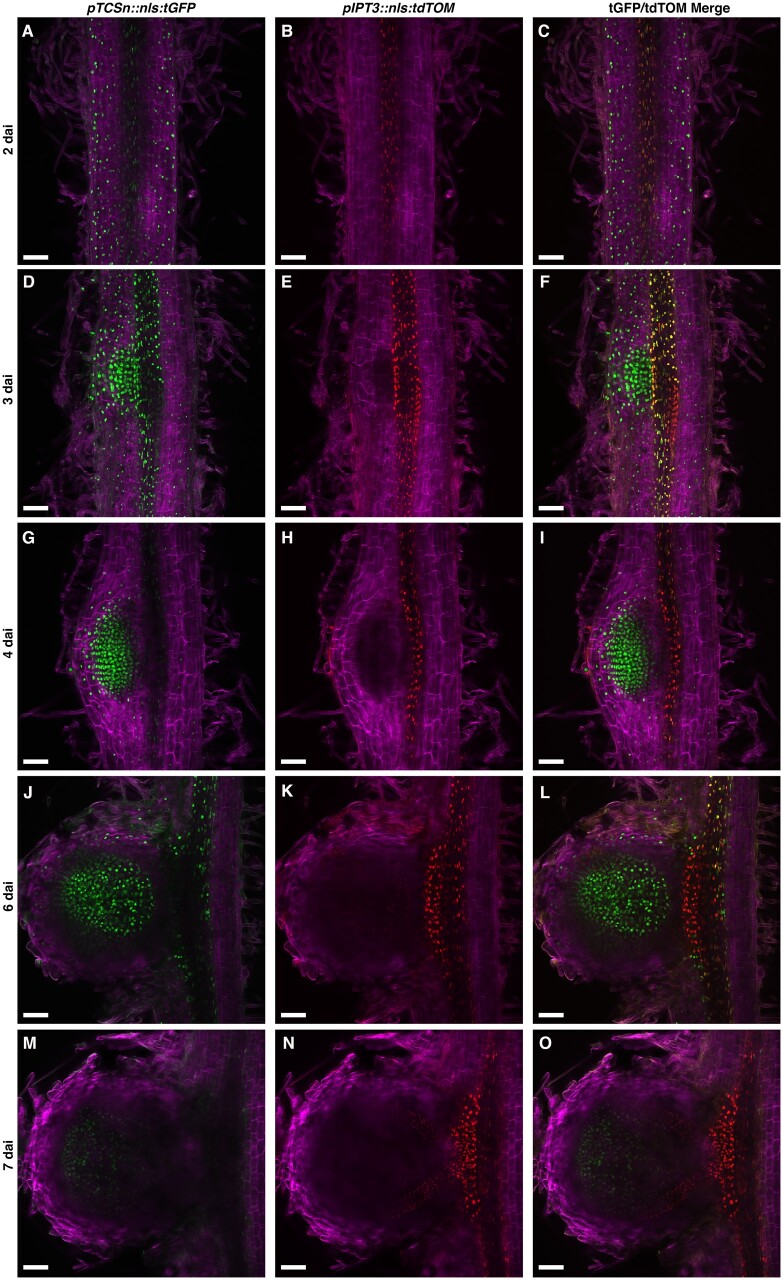
*IPT3* expression is induced in the stele at the base of the nodule primordium during the first cortical cell divisions. A–C, *pTCSn::nls:tGFP* and* pIPT3::nls:tdTOMATO* activities in the susceptible zone of transgenic root at 2  dai of *S. meliloti.* D–I, *pTCSn::nls:tGFP* and* pIPT3::nls:tdTOMATO* activities in different developmental stages of nodule primordium at 3 (D–F) and 4 dai (G–I). J–O, *pTCSn::nls:tGFP* and* pIPT3::nls:tdTOMATO* activities in different developmental stages of mature nodules at 6 (J–L) and 7 dai (M–O). Green and red represent fluorescence signals emitted by tGFP and tdTOMATO, respectively. Scale bar: 100 µm.

To obtain further insights into the role of *IPT3* during nodule organogenesis, we investigated *IPT3* expression by monitoring the accumulation of the tdTOMATO fluorescent protein driven by the *IPT3* promoter in a time-course experiment. This strategy allowed us to monitor *IPT3* expression and CK signaling simultaneously after the inoculation with *S. meliloti* through live imaging in *M. truncatula* transgenic roots. To achieve this goal, we cloned the *IPT3* promoter in frame with the tdTOMATO fluorescent protein fused to a nls peptide, resulting in the *pIPT3::nls:tdTOMATO* construct. Before inoculation with rhizobia, *IPT3* expression and CK response overlapped at the root stele (Supplemental Figure S6, A–C). At 24 h after *S. meliloti* inoculation, *IPT3* expression was similar to the control treatment and still localized in the vasculature (Supplemental Figure S6, D–F). In contrast, the CK response was observed in the epidermis and outer cortical cells of the SZ, as described above (Supplemental Figure S6, G–I). At 2 dai, CK response expanded to most of root cortical layers of the SZ, while *pIPT3::nls:tdTOMATO* activity was still mainly found in the stele with a similar expression level to that of the control and 24 hai (Figure 4, A–C; Supplemental Figure S6, E and H). These results suggest that IPT3 is not involved in the activation of CK signaling during the early symbiotic interaction (48 hai).

At 3 dai, nodule primordium was initiated, and CK response was mainly localized to the dividing cortical cells and stele (Figure 4D). *IPT3* expression was strongly induced in the stele at the base of dividing cortical cells of the nodule primordium (Figure 4E). This result is consistent with prior reports of the induction of the *IPT3* 72 h after *S. meliloti* inoculation (Supplemental Figure S3; Schiessl et al., 2019). The induction of the CK response and *IPT3* expression overlapped at the root vasculature and the base of the nodule primordium (Figure 4F). At 4 dai, the CK response extended to more cortical cell layers of the developing nodule primordium (Figure 4G), whereas *IPT3* expression levels remained high but localized in the stele below the dividing cortical cells (Figure 4H), showing lower overlap with CK signaling in the stele (Figure 4I). At 6 dai, the CK response was mainly localized at the central zone of the nodule (Figure 4J), while *IPT3* was expressed in the stele at the base of the nodule (Figure 4K). At this stage, the overlap between the CK response and *IPT3* expression declined, with each one showing a specific spatial pattern (Figure 4L). At 7 dai, the CK response was mainly localized to the apical part of the nodule (Figure 4M) and *IPT3* expression was detected in the developing nodule vasculature (Figure 4N), in line with the *pIPT3::GUS* activity in nodule vascular bundles recently reported (Azarakhsh et al., 2020). At this stage, the CK response was restricted to the nodule meristem, and *IPT3* expression was localized at the base of the nodule and vascular bundles (Figure 4O). Together, these results suggest that the *IPT3* induction at the base of the nodule primordium contributes to the biosynthesis of CK, which in turn triggers CK signaling during nodule organogenesis.

### *IPT3* expression is activated in the pericycle and pericycle-derived cells during the indeterminate nodule development

**Figure 5:**
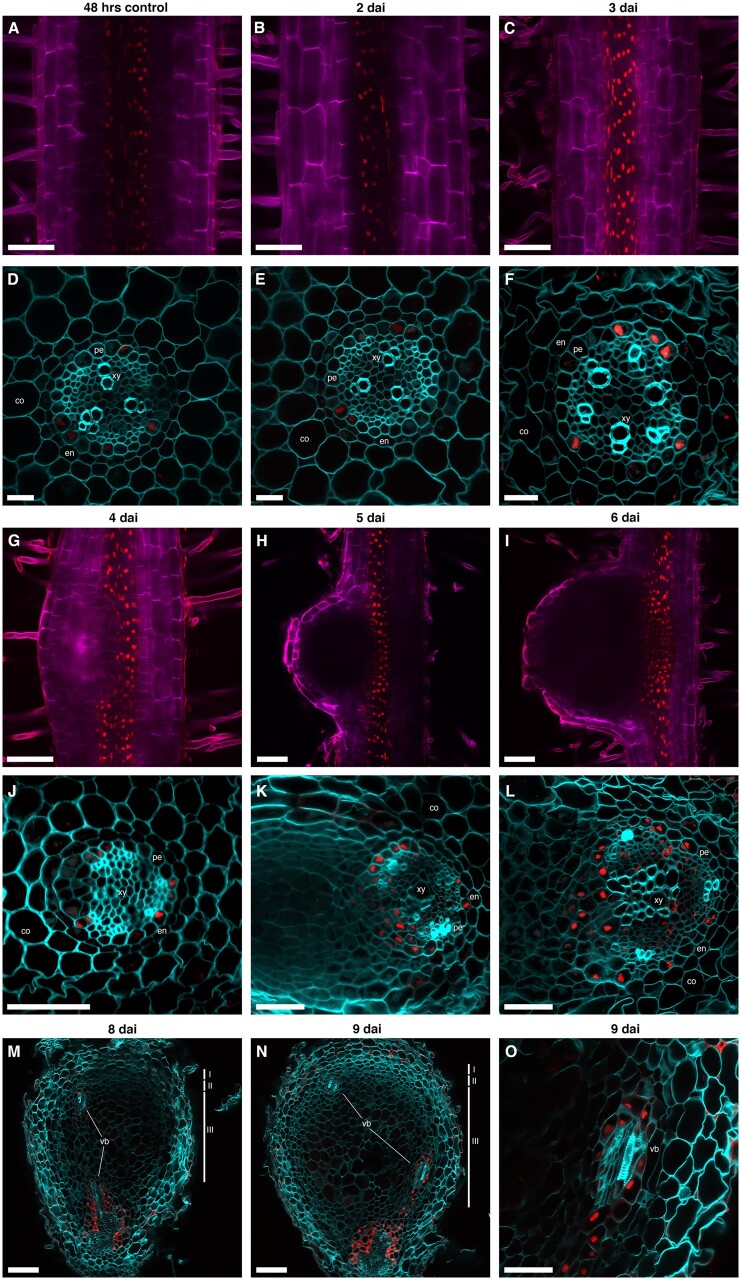
*IPT3* expression is induced in the pericycle and pericycle-derived cells during indeterminate nodule development. A–C, *pIPT3::nls:tdTOMATO* activity in the susceptible zone and (D–F) SZ vibratome cross-sections in the wild-type background, after *S. meliloti* inoculation and in the control. Scale bar: 100 µm (A–C) and 20 µm (D–F). G–I, *pIPT3::nls:tdTOMATO* activity in developing nodules and (J–L) developing nodules vibratome cross-sections in the wild-type background. Scale bar: 100 µm (G–I) and 50 µm (J–L). (M–O) *pIPT3::nls:tdTOMATO* activity in vibratome cross-sections of mature nodules in the wild-type background. Scale bar: 100 µm (M and N) and 50 µm (O). Red represents the fluorescence signal emitted by the tdTOMATO, while the magenta and cyan represent the fluorescence emitted by calcofluor white stain. M and N, I, II, III indicate meristem, infection, and nitrogen fixation zones, respectively. Cortex (co), endodermis (en), pericycle (pe), xylem (xy), and nodule vascular bundles (vb).

To precisely characterize the cell type-specific activation of *IPT3* expression, we monitored the *pIPT3::nls:tdTOMATO* activity in cross-sections of roots inoculated with *S. meliloti*. *pIPT3::nls:tdTOMATO* activity was induced at 3 dai, based on the observation of a higher fluorescence signal of the tdTOMATO protein compared with the control and roots at 2 dai, confirming the transcriptional activation of *IPT3* at 3 dai described above (Figure 5, A–C). The observation of the fluorescence in cross-sections indicated that *IPT3* is induced in the pericycle at 3 dai (Figure 5, D–F). Later in nodule development, *pIPT3::nls:tdTOMATO* activity is localized at the base of nodule primordia, in the stele (Figure 5, G–I). Cross-sections of nodule primordium revealed that *IPT3* expression was restricted to the pericycle and the pericycle-dividing cells at 4 dai (Figure 5J). At 5 and 6 dai, *pIPT3::nls:tdTOMATO* activity was still found in the pericycle, but also in the new cell layers likely derived from the pericycle-dividing cells (Figure 5, K and L). At 8 and 9 dai, *pIPT3::nls:tdTOMATO* activity was detected in the cells surrounding the developing vascular bundles of nodules (Figure 5, M–O). We also explored the *pIPT3::nls:tdTOMATO* activity during lateral root development. We found that *IPT3* expression is restricted to the stele of the primary root during the formation of LRP with a similar fluorescence intensity to the one observed in the stele of the primary root with no LRP (Supplemental Figure S7, A and B). In a fully emerged lateral root, *pIPT3::nls:tdTOMATO* activity was detected in the newly formed stele (Supplemental Figure S7C). Cross-sections showed that *IPT3* is expressed in the pericycle and the cells surrounding the developing vasculature of a recently formed lateral root (Supplemental Figure S7, D and E). Accordingly, *IPT3* expression is activated after 72 h during lateral root formation, when the lateral root fully emerges and the vasculature is already visible, and not in LRP (Supplemental Figure S7F; Schiessl et al., 2019). These results show that *IPT3* gene expression induction is restricted to the pericycle and the pericycle-derived cells during the indeterminate nodule development of *M. truncatula*.

### *IPT3* is required for the induction of symbiotic genes during nodule initiation

**Figure 6:**
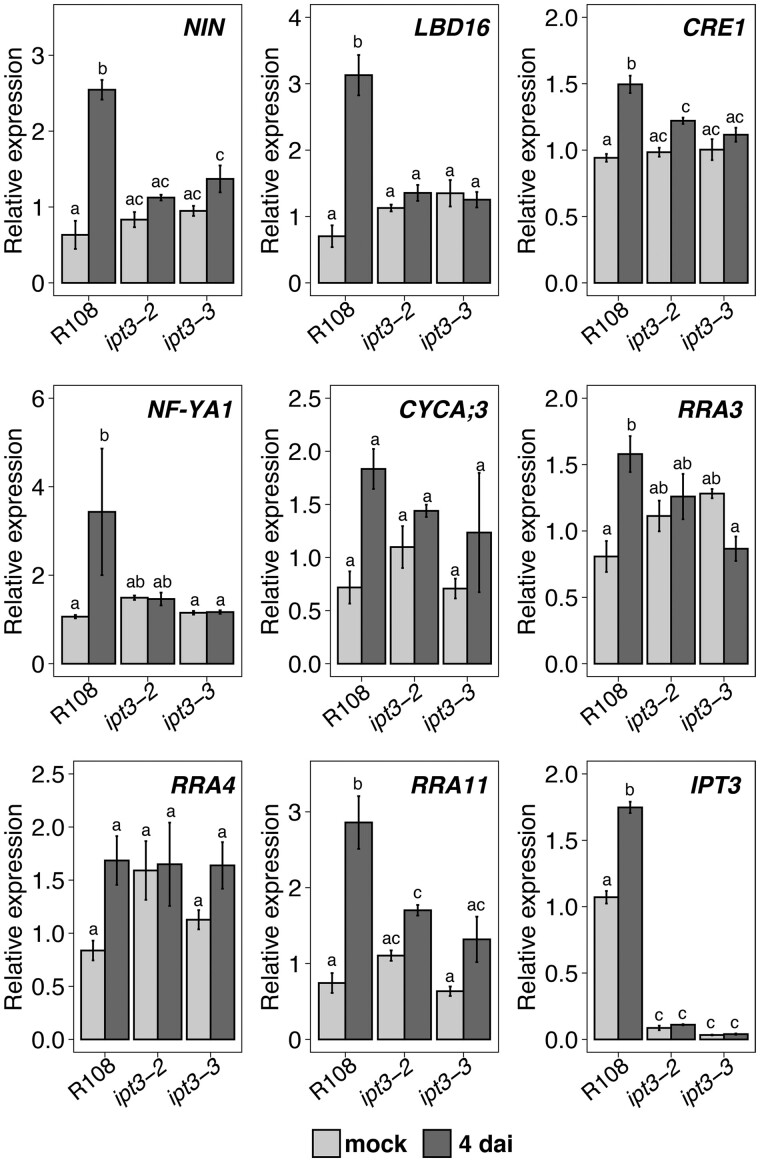
Rhizobia-dependent induction of nodulation regulators and CK signaling genes is impaired in *ipt3* loss-of-function mutants. RT-qPCR analyses of nodulation regulators (*NIN, LBD16*, and *NF-YA1*), cell cycle (*CYCA;3*), and CK signaling genes (*CRE1, RRA3, RRA4*, and *RRA11*) after mock treatment or at 4 dai in wild-type, *ipt3-2*, and *ipt3-3* mutants. Values indicate means ± se of fold-changes of three biological replicates (*n* = 3). *P*-values were calculated by ANOVA followed by Tukey’s post hoc test. Groups of different significance (*P* < 0.05) are indicated with different letters.

Nodule initiation is dependent on crucial regulators that promote cortical cell division, including the CK receptor CRE1, the transcription factor NIN, and its targets *LBD16* and* NF-YA1* (Gonzalez-Rizzo et al., 2006; Plet et al., 2011; Laporte et al., 2014; Vernié et al., 2015; Schiessl et al., 2019). The *ipt3* mutants show impairment of nodule development (Figure 3D), suggesting that the biosynthesis of CK precursor by IPT3 is required to activate CK-induced positive regulators of nodule development. To test this hypothesis, we compared expression of these essential regulatory genes between the wild-type and two *ipt3* mutant lines, *ipt3-2* and* ipt3-3*. The *ipt3-1* mutant was excluded due to the previously mentioned dwarf phenotype. Three-day-old *M. truncatula* plants were inoculated with *S. meliloti*, and the SZ of the root was harvested at 4 dai for gene expression analyses. We found that expression of *NIN*, *LBD16*, and *NF-YA1* was upregulated in wild-type plants compared to noninoculated control plants. In contrast, in the *ipt3* mutants, these genes were not significantly induced (Figure 6), indicating that the transcriptional activation of positive regulators of nodulation requires IPT3 at 4 dai. *CYCLINA;3* (*CYCA;3*), a CK-induced gene and a cell division marker during nodule initiation (Schiessl et al., 2019), did not show significant induction either in the wild-type plants or *ipt3* mutants with respect to the control treatment (Figure 6). Besides *CRE1*, we found significant transcriptional induction of CK signaling genes, such as *RRA3* and* RRA11*, which was affected in *ipt3* mutants compared to the control (Figure 6), indicating IPT3 is required for the activation of CK signaling during nodule initiation. On the contrary, *RRA4* was not induced in wild-type and *ipt3* mutants by rhizobia compared to the control (Figure 6). *IPT3* was upregulated in wild-type plants at 4 dai with respect to the control (Figure 6), consistent with the rhizobia-induced expression pattern observed in previous work and with the visual reporter (Supplemental Figure S3; Schiessl et al., 2019; Figure 5). To better evaluate the impact of IPT3 on CK biosynthesis and signaling during nodulation, we examined *pTCSn::nls:tGFP* activity in wild-type and *ipt3-3* mutant roots, after *S. meliloti* inoculation. As expected, the *ipt3-3* mutant showed lower number of nodules. However, the spatiotemporal characterization of CK sensor showed that CK signaling follows the same pattern in wild-type and *ipt3-3* mutant roots (Supplemental Figure S8), indicating that normal CK signaling occurs in the *ipt3-3.* These results suggested that rhizobia-induced *IPT3* expression contributes to CK biosynthesis, which promotes transcriptional activation of positive nodule development regulators. However, the similar CK signaling pattern in wild-type and *ipt3-3* mutant nodules suggests that other *IPT* genes are induced by rhizobia (Supplemental Figure S3) and can semi-redundantly participate in CK biosynthesis during nodule development.

## Discussion

In this study, we developed a tGFP-based *TCSn* reporter that allowed us to perform a live imaging tissue-specific time course, with a high temporal resolution of rhizobia-induced CK signaling. This reporter system allowed us to gain further insights into the CK signaling induction during rhizobia perception and nodule formation in *M. truncatula*. Our data indicate that the activation of CK signaling occurs in multiple discrete stages, initially in the epidermis of the root SZ and expanding across the cortex during the first 48 h. After 48 h, this widespread CK signaling activation disappears, giving way to the second wave of CK signaling activation in the cortex, localized in the specific cells that will give rise to the nodule primordium. The characterization of *ipt3* mutants suggests that IPT3 contributes to the CK biosynthesis that triggers this second wave of signaling activation.

**Figure 7:**
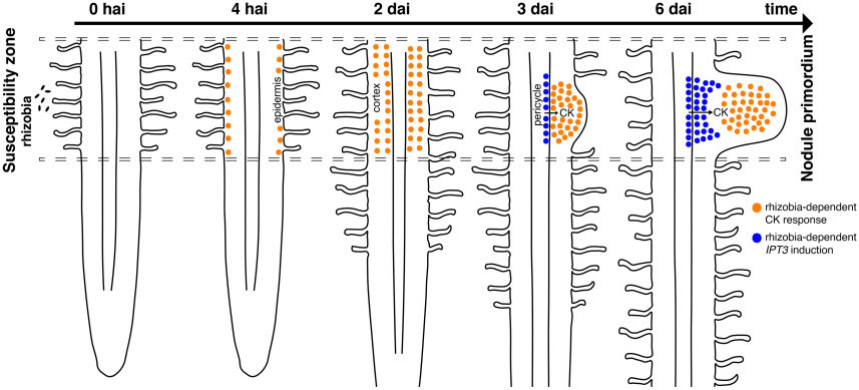
Schematic representation of the spatiotemporal regulation of CK response induced by rhizobia and the proposed function of IPT3 during indeterminate nodule development in *M. truncatula.* At 4  hai, CK signaling activation starts in the epidermal cells and progresses to most of the cortical cell layers within 48 hai. At 3 dai, CK signaling is activated and localized in dividing cortical cells and *IPT3* expression is induced in the pericycle. At 6 dai, the CK response is localized in the central zone of nodule primordium and *IPT3* is expressed in the cells derived from cell division of pericycle and surrounding cells of the nodule developing vasculature.

It has been proposed recently that the *MtABCG56* transporter, which is transcriptionally induced between 6 and 24 h after the LCO treatment, exports bioactive CKs from the root epidermis to the cortex, promoting CRE1-dependent cortical CK responses (Jarzyniak et al., 2021). In agreement with this model, we observed that CK signaling was activated at 24 hai in the outer cortical cells (Figure 2C), and it extends to most of cortical cell layers at 48 hai in the SZ (Figure 2D). Thus, *MtABCG56*-dependent CK transport from epidermis to cortex may contribute to the first wave of CK signaling activation in the cortex (Jarzyniak et al., 2021; Figure 7). These results are consistent with CK activation patterns previously observed using *pTCSn::GUS*, showing GUS activity localized in the epidermis and outer cortical cells at 8 hai and the inner cortical cells at 72 hai, together with in situ hybridization data which detected expression of *RRA4* widely in the root cortex at 48 hai (Vernié et al., 2015; Jardinaud et al., 2016). Here, a live imaging time course allowed us to precisely elucidate the timing of the CK signaling activation pattern observed during the early symbiosis interaction. In contrast, in *L. japonicus*, CK signaling activation in cortical cells precedes epidermal CK responses (Held et al., 2014; Reid et al., 2017). These results suggest that the spatiotemporal CK signaling activation may differ between determinate and indeterminate nodulating species. The difference in rhizobia-triggered CK signaling patterns between these species highlights the need for high-resolution tissue-specific characterization of the CK responses in other legumes.

The second wave of CK signaling activation in the cortex requires de novo CK biosynthesis. It has been reported that CK biosynthesis genes, such as *LOG1*, *LOG2*, and *IPT3*, are expressed in the nodule primordium of *M. truncatula* and that their expression is promoted by KNOX3 (Mortier et al., 2014; Azarakhsh et al., 2020). By monitoring the *pIPT3::nls:tdTOMATO* and the CK sensor simultaneously, we resolved the spatiotemporal pattern of *IPT3* expression and its interaction with the CK signaling during the indeterminate nodule development in *M. truncatula*. We found that *IPT3* is induced in the stele and adjacent cells to the first dividing cortical cells at 3 dai (Figure 4E), overlapping with CK signaling activation at the base of the nodule primordium (Figure 4F). Cross-sections of inoculated roots and developing nodules showed that *IPT3* is induced in the pericycle and the pericycle-derived cells (Figure 5), suggesting that CK biosynthesis in the pericycle by IPT3 promotes cell division. This is unlike prior reports of *pIPT3::GUS* activity in the central zone of the nodule primordium 3 d postinoculation (Azarakhsh et al., 2020). This discrepancy may be explained by a higher sensitivity of the GUS reporter, which could reveal the promoter activity even with very low expression levels. This outcome might also be derived from the diffusion of the GUS reaction product to adjacent cells, especially when extended incubation periods are required. The *ipt3-3* mutant complementation with the *pIPT3::IPT3* construct suggests the promoter region chosen in this study contains the regulatory elements required for the proper expression of *IPT3*. Similar to what was reported with *pIPT3::GUS* 7- to 12-d postinoculation (Azarakhsh et al., 2020), we found that *IPT3* is expressed in those cells surrounding the vascular bundles of the nodules (Figure 5, J–L). Our exploration of *pIPT3::nls:IPT3* activity during lateral root development indicates that *IPT3* expression remains restricted to the stele during LRP formation and only later is activated in the newly formed stele of a fully emerged lateral root (Supplemental Figure S7). Cross-sections of young lateral roots showed that *IPT3* is expressed in the pericycle and developing vasculature (Supplemental Figure S7). Thus, *IPT3* is transcriptionally activated during nodule primordium development but not in LRP. Accordingly, CK responses appear higher in a nodule primordium compared to an LRP (Supplemental Figure S2). The identity of a nodule may be determined by a higher CK/auxin ratio in the nodule primordium than an LRP, as it was observed in soybean (Fisher et al., 2018). *IPT3*-specific activation in the pericycle may contribute to this higher CK/auxin ratio and consequently determine nodule identity.

IPT enzymes catalyze the formation of iP riboside 5′-diphosphate or iP riboside 5′-triphosphate, which are precursors for bioactive CK biosynthesis by LOG family enzymes (Sakakibara, 2006). In *M. truncatula*, *IPT* genes belong to a small gene family with six members, *MtIPT1-5* and *MtIPT9,* whose gene expression is altered by *S. meliloti* inoculation (Supplemental Figure S3; Schiessl et al., 2019). As CK signaling differentially regulates both root colonization and nodule formation, it is expected that CK biosynthesis genes present different tissue-specific expression patterns in response to rhizobia. For example, *IPT2* and* LOG3* are induced in the epidermis after the LCO treatment or *S. meliloti* inoculation (Jardinaud et al., 2016; Jarzyniak et al., 2021), whereas *LOG1* and* LOG2* are expressed in the central zone of developing nodule primordia, overlapping with our observations of the CK signaling activation (Mortier et al., 2014; Azarakhsh et al., 2020). Functional characterization of the other IPTs and their cell-type expression analysis will be critical to understanding the complete landscape of the CK biosynthesis during nodulation. Our observations suggest that the activation of *IPT3* in the pericycle at 3 dai, likely in cooperation with other *IPT* genes (Supplemental Figure S3), provides the substrate for LOG family enzymes to synthesize bioactive CKs which promote nodule primordium development (Kurakawa et al., 2007). Accordingly, *LOG1* RNAi plants show lower number of nodules than control plants in *M. truncatula* (Mortier et al., 2014), similarly to the *ipt3* loss of function mutants. The same effect has been reported in *L. japonicus IPT3* RNAi plants, which produced fewer ITs and nodules than wild-type (Chen et al., 2014).

It has been proposed that *LjIPT3* also participates in the generation of shoot-derived CK precursors, which are involved in the autoinhibition of nodulation (AON) mechanism in *L. japonicus* (Sasaki et al., 2014). Whether *IPT3* participates in AON in *M. truncatula* remains an open question. *IPT3* expression is activated in the shoot at 7 dai in *M. truncatula* in a SUPER NUMERARY NODULES (SUNN)-dependent manner (Azarakhsh et al., 2018). We found that rhizobia-dependent *IPT3* activation in the root occurs at 3 dai in the pericycle (Figure 5F), coinciding with the timing of AON induction (Kassaw et al., 2015). These findings suggest that IPT3-derived CKs may be involved in different mechanisms in shoots and roots. The different timing of *IPT3* activation in different organs could explain its dual role during nodulation in *L. japonicus* and *M. truncatula*.

Rhizobia-dependent transcriptional activation of CK-induced positive regulators of nodule development, such as *NIN*, *CRE1*, *LBD16*, and *NF-YA1*, was affected in *ipt3* mutants at 4 dai (Figure 6). However, the tissue-specific temporal characterization of the CK sensor in *ipt3-3* mutant reveals that CK signaling occurs in the epidermis, cortex, and developing nodules (Supplemental Figure S8). This observation indicates that the nodules formed in *ipt3* mutants present the same CK signaling activation as the wild-type plants. *IPT1*, which shows a marked transcriptional activation in response to rhizobia inoculation (Supplemental Figure S3), may provide the substrate to promote CK production and consequently CK signaling activation in developing nodules of *ipt3-3* mutant. RT-qPCR analyses of CK signaling genes showed a substantial overall reduction of gene expression (Figure 6), according to the lower number of nodules in *ipt3* mutants (Figure 3).

Together, these results suggested that de novo CK precursor synthesis is required for the CK-mediated induction of key nodule development regulators and the proper nodule development. We propose a high-resolution model for the tissue-specific temporal activation pattern of CK signaling during indeterminate nodule development in *M. truncatula* (Figure 7). Furthermore, we show that IPT3 contributes to CK biosynthesis, which in turn promotes the expression of positive regulators of nodule development, such as *NIN* and* CRE1*.

## Materials and methods

### Plant material and growth conditions

*Medicago truncatula* R108 is the wild-type background for experiments involving the *Tnt1* mutant lines; Jemalong A17 was used for all other assays. Seeds were scarified for 8 min in sulfuric acid and sterilized for 4 min in bleach (12% [v/v] sodium hypochlorite). After rinsing with sterilized water, seeds were sown on 1% (w/v) agar plates supplemented with 1 μM gibberellic acid and stored at 4°C for 3 d before incubating overnight at 24°C in the dark. Germinated seedlings were transferred to square plates (22.5 × 22.5 cm) containing modified Fahräeus medium (Boisson-Dernier et al., 2001) supplemented with 15 mM NH_4_NO_3_ and grown vertically at 24°C under long-day conditions in a growth chamber (16-h light/8-h dark; 150 μmol m^−2^ s^−1^ light intensity).

### Cloning

The Golden Gate MoClo and MoClo Plant toolkits (Addgene) were used to generate all constructs described in this work (Engler et al., 2014). The *TCSn* promoter DNA sequence (Zürcher et al., 2013) comprising overhangs was synthesized by Synbio Technologies and cloned in frame with a nls (Supplemental Table S1), turbo GFP (Supplemental Table S1), and tomato (*Solanum lycopersicum*) ATPase terminator (Supplemental Table S1) in the level 1 vector pICH47811 (Supplemental Table S1). To amplify the *IPT3* promoter (gene ID version 4: Medtr1g072540; version 5: MtrunA17Chr1g0185751) genomic DNA (gDNA) extraction was performed using 3-d-old *M. truncatula* A17 seedlings, as previously described (Triozzi et al., 2021). A genomic fragment of 2,312-bp upstream of the *IPT3* coding sequence (CDS), including the 5′-untranslated region (UTR) was amplified using primers containing overhangs (Supplemental Table S2) from *M. truncatula* A17 gDNA. About 100 ng of gDNA was used in a PCR using Phusion High-Fidelity DNA Polymerase (New England BioLabs, Ipswich, MA, USA). The PCR product was separated in a 1% (w/v) agarose gel by electrophoresis. The *IPT3* promoter DNA sequence was extracted and purified from the gel using Monarch DNA Gel Extraction Kit (New England Biolabs), and Sanger sequenced at Genewiz (South Plainfield, NJ). The *IPT3* promoter was cloned in frame with *IPT3* CDS containing overhangs (Synbio Technologies) and 35S terminator in the level 1 vector pICH47822 (Supplemental Table S1). The tdTOMATO CDS was amplified using primers containing overhangs (Supplemental Table S2). The *IPT3* promoter was cloned in frame with nls, tdTOMATO CDS, and 35S terminator (Supplemental Table S1) in the level 1 vector pICH47802 (Supplemental Table S1). For complementation of *ipt3-3* mutant, level 1 pICH47822-*pIPT3::IPT3::t35S* was cloned together with pICH47802-*p35S::ER:tdTOM::tNOS* and pICH47811-*pTCSn::nls:tGFP* in the final plant expression vector pAGM4673 (Supplemental Table S1). For spatiotemporal characterization of the CK sensor, level 1 pICH47811-*pTCSn::nls:tGFP::tATPase* was cloned together with the pICH47802-*p35S::ER:tdTOM::tNOS* selection marker (Triozzi et al., 2021) in pAGM4673. For simultaneous live imaging, pICH47811-*pTCSn::nls:tGFP* was cloned together with pICH47802-*pIPT3::nls:tdTOMATO::t35S* in pAGM4673.

### Genotyping of *Tnt1* insertion lines

*Medicago* *truncatula* R108 *Tnt1* transposon insertion lines utilized in this research project, which are jointly owned by the Centre National De La Recherche Scientifique, were obtained from the Noble Research Institute. Three different *Tnt1* transposon insertion lines, namely NF5762 (*ipt3-1*), NF3757 (*ipt3-2*), and NF4651 (*ipt3-3*), were genotyped by PCR using *Tnt1*-specific primers (Cheng et al., 2011) combined with *MtIPT3* gene-specific primers encompassing the insertions (Supplemental Table S2). The expression of *MtIPT3* in homozygous plants was tested by RT-qPCR to confirm they were knockout mutants of the *MtIPT3* gene (Supplemental Table S2).

### Complementation of *ipt3-3* mutant

One-day-old wild-type and *ipt3-3* seedlings were transformed with *Agrobacterium rhizogenes* MSU440, as previously described (Boisson-Dernier et al., 2001). Three-week-old composite plants showing transgenic roots were transferred to growth pouches (https://mega-international.com/tech-info/) containing Modified Nodulation Medium (Chakraborty et al., 2021). The plants were acclimated for a week and then inoculated with *phemA::lacZ S. meliloti* 1021 (Leong et al., 1985). The nutrient medium was replenished every week. Two weeks after inoculation, transformed roots expressing *p35S::ER:tdTOM::tNOS* were selected based on the presence of the tdTOMATO fluorescence and stained for *lacZ* (5 mM potassium ferrocyanide, 5 mM potassium ferricyanide, and 0.08% (w/v) X-gal in 0.1 M PIPES, pH 7) for 4 h at 37°C. The total nodule number from transgenic roots per plant was scored under an Olympus MVX10 fluorescence stereo microscope.

### *Agrobacterium rhizogenes*-mediated transformation and *in vitro* nodulation assay

The constructs described above were introduced into *A. rhizogenes* MSU440 electrocompetent cells and used to generate transgenic roots in *M. truncatula* (Boisson-Dernier et al., 2001). The transgenic roots were selected based on the fluorescence emitted by the *pTCSn::nls:tGFP* construct at the root tip, visualized under an Olympus MVX10 fluorescence stereo microscope. For the *in vitro* nodulation assay time-course experiment, 3-week-old transgenic roots were transferred to Buffered Nodulation Medium (Ehrhardt et al., 1992) supplemented with 0.1 µM AVG (aminoethoxyvinyl glycine hydrochloride; Sigma-Aldrich, St Louis, MO, USA) to reduce ethylene production. After 5 d of acclimation, transgenic roots were treated with a suspension of *S. meliloti* 1021 (OD_600_ = 0.02) supplemented with 3 µM of luteolin to activate LCO production (Sigma-Aldrich) and transgenic roots were collected at different timepoints. To characterize *pTCSn::nls:tGFP* activity in *ipt3-3* mutant, 3-week-old transgenic roots were grown in pouches as described above. After *S. meliloti* 1,021 inoculation, transgenic roots were harvested at different timepoints for live imaging.

### *In vitro* CK treatment and *S. meliloti* inoculation

Three-day-old *M. truncatula* seedlings were transferred to Fahräeus medium supplemented with water (mock treatment) or 1 µM of 6-BAP (Sigma-Aldrich) and maintained under the same growth conditions. Five roots from different seedlings were collected at 1, 8, 24, and 48 h after CK treatment and immediately frozen in liquid nitrogen for RNA extraction. To analyze *pTCSn::nls:tGFP* activity after CK treatment, *M. truncatula* transgenic roots were submerged in a solution of 1 µM 6-BAP for 5 min. After 24 h, transgenic roots harboring the *pTCSn::nls:tGFP* construct were harvested and used for microscopy analysis. For RT-qPCR studies of nodule development regulators and CK signaling genes in wild-type and *ipt3* mutants, germinated seedlings were transferred to nitrogen-free Fahräeus medium and grown under the same conditions described above. Three-day-old seedlings were inoculated alongside the root with 200 µL/root of *S. meliloti* 1021 (OD_600_ = 0.02) resuspended in liquid nitrogen-free modified Fahräeus medium or with Fahräeus medium (mock treatment). Four days after inoculation, root segments from the SZ (3 cm sections from 2 cm above the root tip) were harvested and pooled (10 plants per sample) for RNA extraction.

### Gene expression analysis

RNA extraction was performed as previously described (Chang et al., 1993). RNA samples were digested with DNase I and purified using the RNA Clean & Concentrator-5 kit following the manufacturer’s instructions (Zymo Research, Irvine, CA, USA). RT-qPCR analyses were performed using the Luna Universal Probe One-Step RT-qPCR Kit (New England Biolabs) following the manufacturer’s instructions using 200 ng of total RNA. The *EF1*α gene was used as a housekeeping gene and the average of two technical replicates was obtained to calculate relative gene expression (ΔΔCt method). Primers used for RT-qPCR experiments are listed in Supplemental Table S2.

### Microscopy live imaging

Before microscopy imaging, transgenic roots were placed in a staining solution with 0.1% (w/v) Calcofluor white M2R (Sigma-Aldrich) in PBS 1X (Corning) for 10 min at room temperature and then rinsed with PBS 1X before imaging. A Leica TCS SP5 confocal microscope was used for live imaging. For cross-sectioning, roots and nodules were submerged in a freshly prepared solution of 4% (w/v) formaldehyde in PBS 1X, vacuuming three times for 5 min followed by overnight fixation at 4°C. The fixed roots and nodules were embedded in 6% (w/v) agarose and sectioned (50 μm of thickness) in PBS 1X using a Leica VT1000S vibratome. A confocal microscope Leica TCS SP5 was used for live imaging. At least ten different roots for treatment or time points were analyzed. Images were acquired by a HCX PL APO CS 20.0X0.70 dry and HCX PL APO lambda blue 63.0X1.40 Oil. tGFP was excited with 488-nm laser (Argon) set at 80% of intensity and the emission was collected at 497–557 nm. tdTOMATO was excited with 514-nm (Argon) and 543-nm lasers (HeNe 543) set at 80% of intensity and the emission was collected at 570–643 nm. Calcofluor white M2R was excited with 405-nm Diode laser set at 40% of intensity and the emission was collected at 450–480 nm. Fluorescent signals are presented with green (tGFP), red (tdTOMATO), and magenta or cyan (calcofluor white M2R). For DNA staining, transgenic roots were incubated with 5 μg/mL DAPI in PBS 1× for 10 min at room temperature and then rinsed with PBS 1X before imaging. DAPI was excited with 405-nm Diode laser set at 40% of intensity and emission was collected at 450–480 nm. The confocal gain and pinhole were set to 700 and 60 mm, respectively, and the images were acquired at 1,024 × 1,024 pixels resolution.

### *In vivo* nodulation assay

For *in vivo* nodulation assay, seedlings were germinated as described above. Then, plants were grown in pots (9 × 9 × 9 × cm) containing pre-sterilized calcined clay, Turface (Profile Products, Buffalo Grove, IL, USA), and sand (2:2 v/v), where Turface was placed at the bottom and on the top of a layer of sand. Plants were watered with modified Fahräeus medium supplemented with 0.5 mM of NH_4_NO_3_ and covered with a lid. After 1 week of acclimation, Fahräeus medium supplemented with 0.5 mM of NH_4_NO_3_ was removed entirely from the tray and replaced with a nitrogen-free modified Fahräeus medium. Plants were treated by pouring 10 mL of *S. meliloti* 1,021 suspension (OD_600_ = 0.02) into each pot. Plants were watered using nitrogen-free modified Fahräeus medium every 2–3 d. After 2 weeks of inoculation, nodule number was assessed by inspecting plant roots under an Olympus MVX10 fluorescence stereo microscope. After counting nodules, roots and shoots for each individual were separated and dried in paper bags. After 48h of drying at 65°C, root and shoot dry weight were recorded.

### Nodule sectioning

Plants were grown in growth pouches as described above and treated with *phemA::lacZ S. meliloti* 1021 (Leong et al., 1985). After 2 weeks, nodules were stained for *lacZ* as described above overnight at 37 °C. Next, nodules were fixed in 4% (w/v) formaldehyde in PBS 1X (Corning), by vacuuming three times for 5 min followed by 4 h at 4°C. The fixed nodules were embedded in 6% (w/v) agarose and sectioned (50 μm of thickness) in PBS 1X using a Leica VT1000S vibratome. Images of nodule sections were taken with a Zeiss Axioplan 2 microscope attached to a QImaging Retiga EXi Fast 1394 camera.

### Acetylene reduction assay

Seedlings were germinated as described above then grown in pots (6 × 2.5 × 2.5 cm) containing 4:1 pre-sterilized turface and sand. After 5 d of growth, 5 mL of *phemA::lacZ S. meliloti* 1021 (OD_600_ = 0.02) was added to each pot. Plants were fertilized weekly with 5 mL of Plant Prod 0-15-40 medium (nitrogen free, pH 6.8; Master PlantProd Inc.; Brampton, Ontario, Canada). Three weeks after inoculation, plants were gently removed from the substrate without washing, and an acetylene reduction assay was performed as described previously (Oke and Long, 1999). After this, plants were stained for *lacZ* as described above to visualize infected nodules, and nodule number counted under a stereo microscope.

### Accession numbers

Accesion numbers of the genes studied in this work can be found in the Medicago Genome version 4.0 available in Phytozome database, as follows: *EF1α* (Medtr1g101870), *IPT1* (Medtr1g110590), *IPT2* (Medtr4g117330), *IPT3* (Medtr1g072540), *IPT4* (Medtr2g022140), *IPT5* (Medtr4g055110), *IPT9* (Medtr2g078120), *RRA3* (Medtr3g088630), *RRA4* (Medtr5g036480), *RRA11* (Medtr8g038620), *CRE1* (Medtr8g106150), *NF-YA1* (Medtr1g056530), *LBD16* (Medtr7g096530), *NIN* (Medtr5g099060), and *CYCA;3* (Medtr3g102530).

## Supplemental data

The following materials are available in the online version of this article.

**Supplemental Figure S1.** *pTCSn::nls:tGFP* emitted fluorescence signal is localized into *M. truncatula* root nuclei.

**Supplemental Figure S2.** *pTCSn::nls:tGFP* activity is higher in nodule primordium than LRP.

**Supplemental Figure S3.** *IPT* expression levels in a time-course experiment after *S. meliloiti* inoculation in *M. truncatula* roots.

**Supplemental Figure S4.** Root and shoot dry weight measurements of wild-type and *ipt3* mutants at 14 dai.

**Supplemental Figure S5.** Nodules of *ipt3* mutants show normal rhizobial colonization and nitrogen fixation capacity.

**Supplemental Figure S6.** *IPT3* expression is not induced after 24 h of *S. meliloti* inoculation and is localized in the stele of *M. truncatula* root.

**Supplemental Figure S7.** *IPT3* is expressed in the stele of LRP and lateral root of *M. truncatula*.

**Supplemental Figure S8.** Rhizobia-dependent CK signaling activation is still occurring in epidermis, cortex, and developing nodules of *ipt3-3* mutant.

**Supplemental Table S1.** List of plasmids used in this study.

**Supplemental Table S2.** List of primers used in this study.

## Funding

This work was supported by the Department of Energy Office of Science Biological and Environmental Research (Grant DE-SC0018247) to M.K. and J.M.A.

##  

*Conflict of interest statement*. None declared.

## Supplementary Material

kiab447_Supplementary_Materials

## Notes

P.M.T., M.K., and D.C. designed the research; P.M.T., T.B.I., Z.P.K., S.C., J.M.A., M.K., and D.C. designed the methodology; P.M.T., T.B.I., H.W.S., and D.C. performed the experiments; T.B.I., Z.P.K., S.C., C.D., and J.M.A. provided material and technical advice. K.S.M. and J.W. supplied plant material. P.M.T., M.K., and D.C. wrote the paper. T.B.I., H.W.S., S.C., K.B., W.P., C.D., and J.M.A. supervised the data analyses and reviewed and edited the paper. M.K. and D.C. supervised the methodology, data analyses, and writing-editing of the paper.

The author responsible for distribution of materials integral to the findings presented in this article in accordance with the policy described in the Instructions for Authors (https://academic.oup.com/plphys/pages/General-Instructions) is: Matias Kirst (mkirst@ufl.edu)
