# Supplementary material for: Spatiotemporal cytokinin response imaging and ISOPENTENYLTRANSFERASE 3 function in Medicago nodule development
Source: Plant Physiol. 2021 Sep 21;188(1):560–75. doi: 10.1093/plphys/kiab447 (PMC8774767; doi:10.1093/plphys/kiab447)
Supplement: kiab447_Supplementary_Materials [file kiab447_supplementary_materials.pdf]

SUPPLEMENTAL MATERIALS

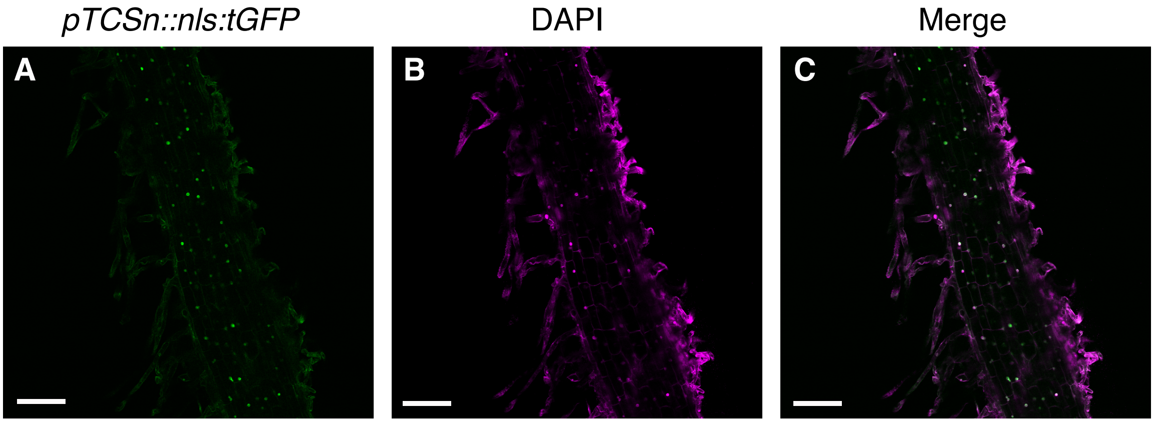

Supplemental Figure S1. *pTCSn::nls:tGFP* emitted fluorescence signal is localized into *M. truncatula* root nuclei. (A) Transgenic root expressing *pTCSn::nls:tGFP* (green) after 24 hours of 6-BAP treatment. (B) Same transgenic root stained with DAPI (magenta) and (C) merged image showing brighter nuclei where tGFP and DAPI signals overlap. Scale bars: 100 µm.

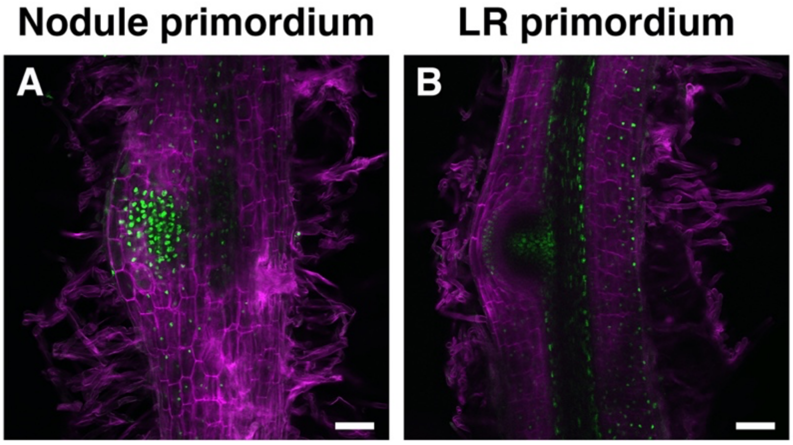

Supplemental Figure S2. *pTCSn::nls:tGFP* activity is higher in nodule primordium than lateral root primordium. *pTCSn::nls:tGFP* activity in (A) nodule primordium and (B) lateral root (LR) primordium. Scale bar: 100 µm. Green and magenta represent fluorescence signals emitted by tGFP and calcofluor white stained, respectively.

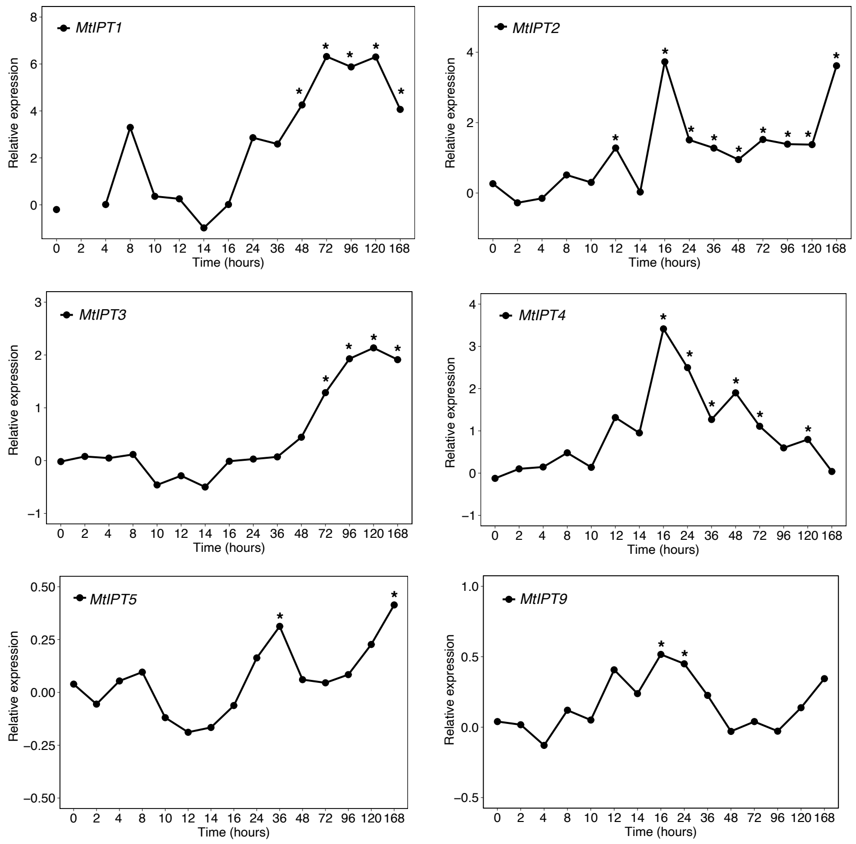

Supplemental Figure S3. *IPT* expression levels in a time-course experiment after *S. meliloiti* inoculation in *M. truncatula* roots. Expression data obtained from Schiessl et al., 2019. Expression values are log2 fold changes. Expression values are log2 fold changes and were calculated using the R package DESeq2 (Love et al., 2014). Asterisk indicates significant expression changes (False discovery rate corrected *P* value < 0.05; n=3). Gene nomencalture is same described by Azarakhsh et al., 2018. *IPT1* (Medtr1g110590), *IPT2* (Medtr4g117330), *IPT3* (Medtr1g072540), *IPT4* (Medtr2g022140), *IPT5* (Medtr4g055110), *IPT9* (Medtr2g078120).

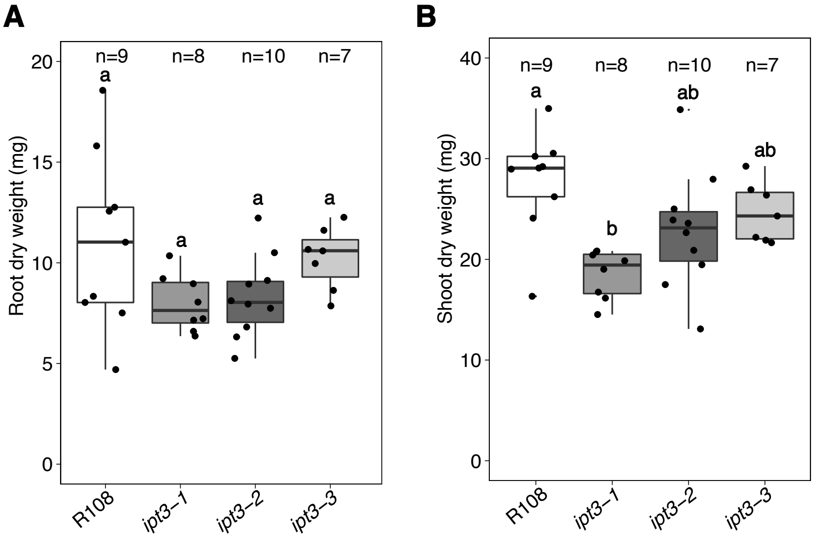

Supplemental Figure S4. Root and shoot dry weight measurements of wild-type and *ipt3* mutants at 14 dai. (A) Root and (B) shoot dry weight measurements of wild-type and *ipt3* mutant plants at 14 dai. Boxplots center line, median; the box extends from the 25th to 75th percentiles; whiskers, 1.5x interquartile range; points out of the whiskers, outliers. Statistical analysis was performed using ANOVA followed by Tukey's post-hoc test. Groups of different significance (P<0.05) are indicated with different letters.

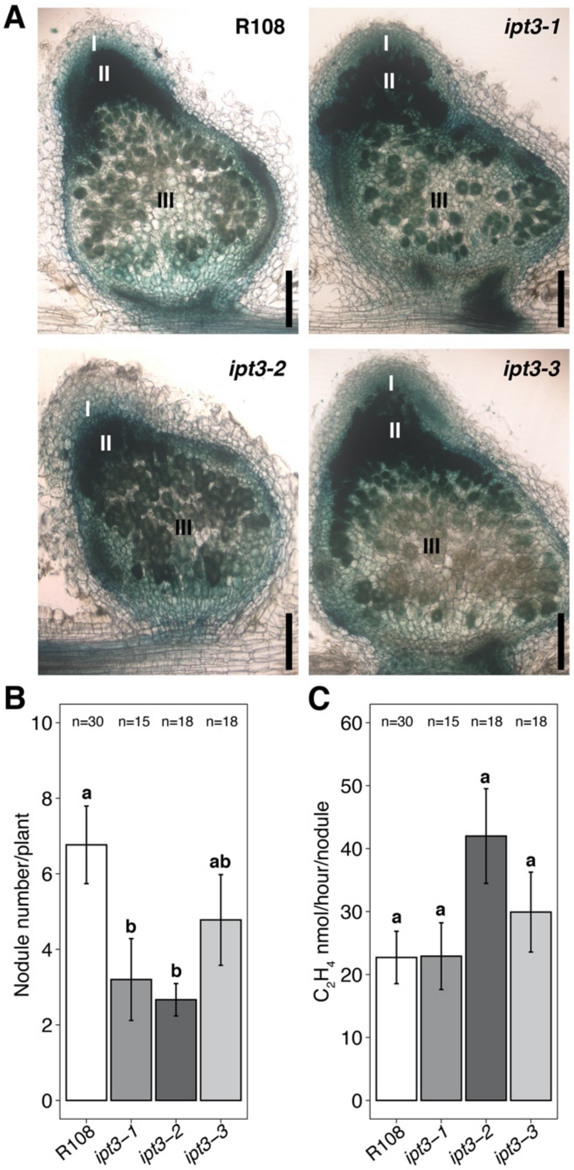

Supplemental Figure S5. Nodules of *ipt3* mutants show normal rhizobial colonization and nitrogen fixation capacity. (A) Cross-sections of wild-type and *ipt3* mutant nodules at 14 days after inoculation (dai) stained with X-gal (blue; bacteria expressing *phemA:lacZ*), showing that all lines develop large cells hosting many symbiosomes. I, II, III indicate meristem, infection, and nitrogen fixation zones, respectively. Scale bar: 250 µm. (B) Number of nodules per plant and (C) amount of acetylene reduced per nodule per hour as a proxy for rate of nitrogen fixation; both at 21 dai. Values are the means ± SE. Statistical analysis was performed using ANOVA followed by Tukey's post-hoc test. Groups of different significance (P<0.05) are indicated with different letters.

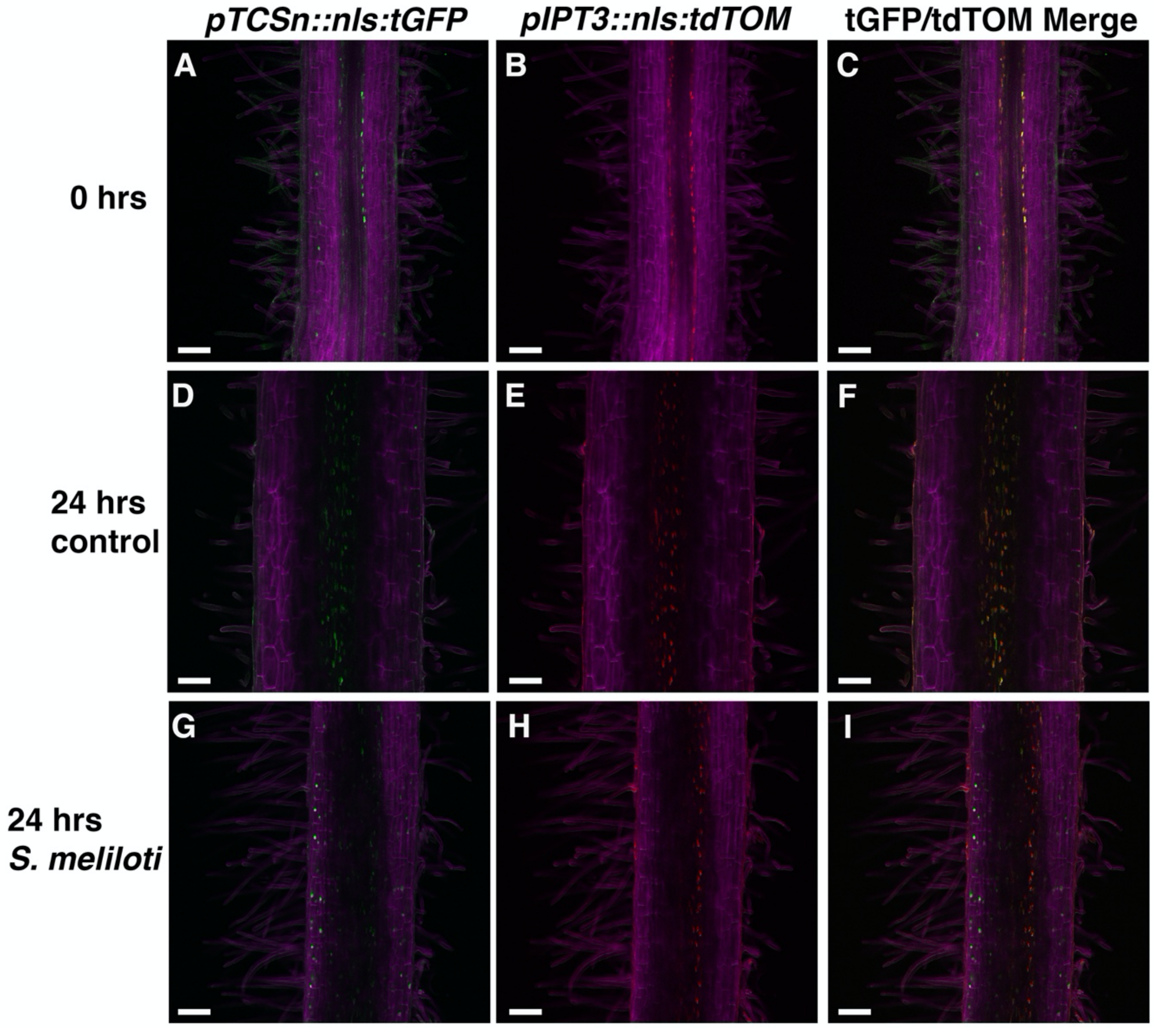

Supplemental Figure S6. *IPT3* expression is not induced after 24 hours of *S. meliloti* inoculation and is localized in the stele of *M. truncatula* root. (A-C) *pTCSn::nls:tGFP* activity (green) and *pIPT3::nls:tdTOMATO* activity (red) and cell walls (calcofluor white stained, magenta) in the stele and from susceptible zone of untreated transgenic root, (D-F) 24 hours after a mock inoculation or (G-I) inoculation with *S. meliloti*. Scale bar: 100 μm.

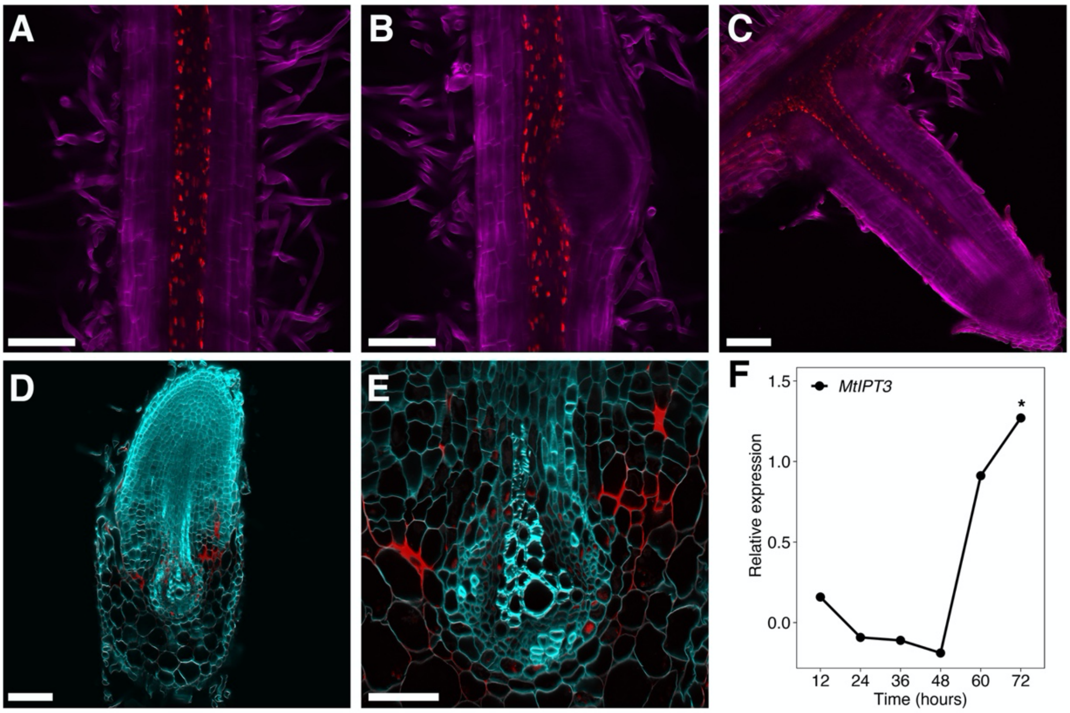

Supplemental Figure S7. *IPT3* is expressed in the stele of lateral root primordium and lateral root of *M. truncatula*. (A, B, C) *pIPT3::nls:tdTOMATO* activity in primary root, lateral root primordia and lateral root. Scale bar: 100 µm. (D, E) Cross-section of lateral root expressing *pIPT3::nls:tdTOMATO*. Scale bar: 100 µm (D) and 50 µm (E). Red and magenta or cyan represent fluorescence signals emitted by tdTOMATO and calcofluor white stained, respectively. (F) *IPT3* expression levels in a time-course experiment during lateral root development in *M. truncatula*. Expression data obtained from Schiessl et al., 2019. Expression values are log2 fold changes and were calculated using the R package DESeq2 (Love et al., 2014). Asterisk indicates significant expression changes (False discovery rate corrected *P* value < 0.05; n=3).

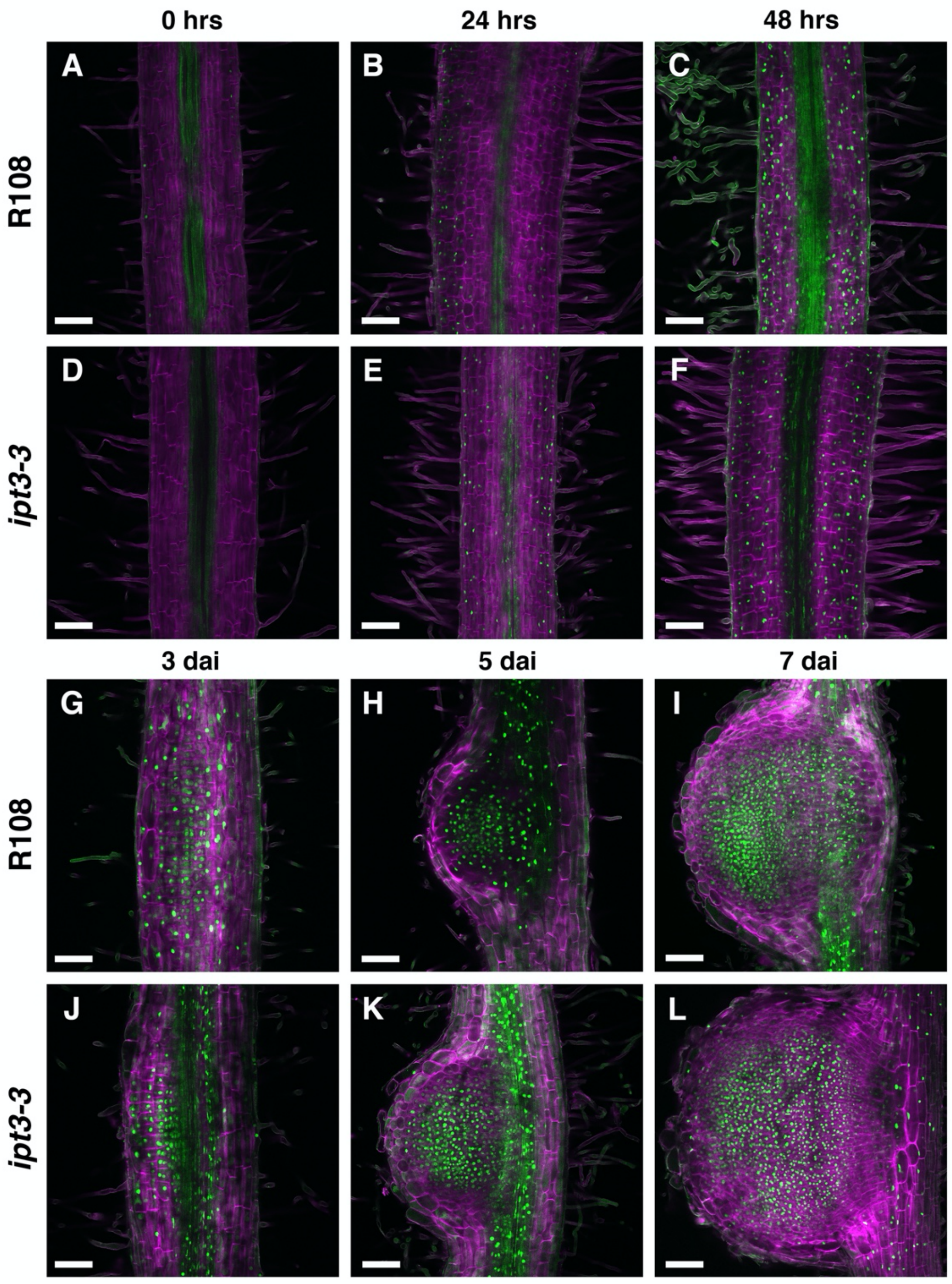

Supplemental Figure S8. Rhizobia-dependent CK signaling activation is still occurring in epidermis, cortex, and developing nodules of *ipt3-3* mutant. (A-C) *pTCSn::nls:tGFP* activitiy in the susceptible zone of transgenic root of wild-type and (D-F) *ipt3-3* mutant after *S. meliloti* inoculation. (G-I) *pTCSn::nls:tGFP* activitiy in different developmental stages of the nodule primordia at 3, 5 and 7 days after inoculation (dai) of transgenic root of wild-type and (J-L)

ipt3-3 mutant. Green and magenta represent fluorescence signals emitted by tGFP and calcofluor white stained, respectively. Scale bar: 100 µm.

Supplemental Table S1. List of plasmids used in this study.

Name	Plasmid	Catalog number	Kit
nuclear localization signal	pAGM5331	50294	MoClo Plant Parts Kit
turbo GFP	pICSL80005	50322	MoClo Plant Parts Kit
Solanum lycopersicum ATPase terminator	pICH71431	50344	MoClo Plant Parts Kit
35S terminator	pICH41414	50337	MoClo Plant Parts Kit
Acceptor level 1 position 1	pICH47802	48007	MoClo Toolkit
Acceptor level 1 position 2	pICH47811	48008	MoClo Toolkit
Acceptor level 1 position 3	pICH47822	48009	MoClo Toolkit
Acceptor level 2	pAGM4673	48014	MoClo Toolkit

Supplemental Table S2. List of primers used in this study.

Primer name	Gene ID	Primer Forward sequence 5' to 3'	Primer Reverse sequence 5' to 3'	Reference
pMtIPT3_nls	Medtr1g072540	GGTCTCAGGAGTTCCTTAATTATTATCTTGAGGAC	GGTCTCTAGTAGATGATGATAAAAATTAATTCAAAG	This study
pMtIPT3_CDS	Medtr1g072540	GGTCTCAGGAGTTCCTTAATTATTATCTTGAGGAC	GGTCTCACATTGATGATGATAAAAATTAATTCAAAG	This study
tdTOMATO		AATGGTCTCAAATGGTGTCCAAGGGCGAAGA	ATTGGTCTCTAAGCTTATTTGTAAAGCTCGTCCATTCCG	This study
MtIPT3_genotyping	Medtr1g07 2540	ACTAGCACAACCTATGCAACCTTAG	ACTCTATATTGAATGGGATTCCAG	This study
Tnt1		TCCTTGTTGGATTGGTAGCC	CAGTGAACGAGCAGAACCTGTG	Cheng et al. 2011
MtIPT3_qPCR	Medtr1g07 2540	TGGAAGAAGATTGTTGCAGAGC	TTGCAGTAGCCATTAGATCACC	This study
MtRRA3_qPCR	Medtr3g088630	ATGGAGCTTGGTTTGGATAGTAG	ACAGGTAACTTTGCATGAAGAATC	This study
MtRRA4_qPCR	Medtr5g036480	ATGCTTTTGTTCCGGGTTTA	CTGCACCTTCCTCCAAACAT	Vernié et al. 2008
MtRRA11_qPCR	Medtr8g038620	TGAGAATCAGTTCCATGTTTTG	TCATTTCATCTTCAACCAAACC	This study

MtCRE1_qPCR	Medtr8g106150	GATGCCTAATGGTTCAACTTCAGTTC	CTGCACACTTCACATCCGCTC	This study
MtEF1α_qPCR	Medtr1g10 1870	GAGGCCATCAGACAAGC	GTCTCAACACGTCCCACAG	This study
MtNFYA1_qPCR	Medtr 1g056530	ATCATCAGACGCAGGCATTCTCG	TCGTGCATATATGGCTTGTTACGC	Schiessl et al. 2019
MtLBD16_qPCR	Medtr7g096530	AGCTCGTATCAGAGACCCTGTG	TGCAAGCATGCTACCTGTTGTTG	Schiessl et al. 2019
MtCYCA;3_qPCR	Medtr3g102530	GCTTCTCCCTCAAACCCTTCA	CGATGAGCATGGATGAAACACC	Schiessl et al. 2019
NIN_qPCR	Medtr5g099060	GGAAGATTGAGAGGGGAAGCTT	GCAATGTGGGGATTTAGAGATT	Marsh et al. 2007

REFERENCES

Cheng X, Wen J, Tadege M, Ratet P, Mysore KS (2011) Reverse Genetics in Medicago truncatula Using Tnt1 Insertion Mutants. *In* A Pereira, ed, Plant Reverse Genetics. Humana Press, Totowa, NJ, pp 179–190

Marsh JF, Rakocevic A, Mitra RM, Brocard L, Sun J, Eschstruth A, Long SR, Schultze M, Ratet P, Oldroyd GED (2007) *Medicago truncatula NIN* Is Essential for Rhizobial-Independent Nodule Organogenesis Induced by Autoactive Calcium/Calmodulin-Dependent Protein Kinase. Plant Physiol **144**: 324–335

Love MI, Huber W, Anders S (2014) Moderated estimation of fold change and dispersion for RNA-seq data with DESeq2. Genome Biol **15**: 550

Schiessl K, Lilley JLS, Lee T, Tamvakis I, Kohlen W, Bailey PC, Thomas A, Luptak J, Ramakrishnan K, Carpenter MD, et al (2019) NODULE INCEPTION Recruits the Lateral Root Developmental Program for Symbiotic Nodule Organogenesis in Medicago truncatula. Current Biology **29**: 3657-3668.e5

Vernié T, Kim J, Frances L, Ding Y, Sun J, Guan D, Niebel A, Gifford ML, de Carvalho-Niebel F, Oldroyd GED (2015) The NIN Transcription Factor Coordinates Diverse Nodulation Programs in Different Tissues of the *Medicago truncatula* Root. Plant Cell **27**: 3410–3424
